# Temperature dependence of the dielectric function and critical points of monolayer WSe_2_

**DOI:** 10.1038/s41598-024-64303-1

**Published:** 2024-06-12

**Authors:** Xuan Au Nguyen, Long V. Le, Suk Hyun Kim, Young Duck Kim, Mangesh S. Diware, Tae Jung Kim, Young Dong Kim

**Affiliations:** 1 Department of Physics, Kyung Hee University, Seoul, 02447 Republic of Korea; 2 Institute of Materials Science, Vietnam Academy of Science and Technology, Hanoi, 100000 Vietnam; 3 Department of Information Display, Kyung Hee University, Seoul, 02447 Republic of Korea; 4 Advanced Research Center, Parksystems Co., Suwon, Republic of Korea; 5 Center for Converging Humanities, Kyung Hee University, Seoul, 02447 Republic of Korea

**Keywords:** Monolayer WSe_2_, 2D-TMDC, Ellipsometry, Temperature dependence, Materials science, Materials for devices, Materials for optics

## Abstract

Monolayer materials typically display intriguing temperature-dependent dielectric and optical properties, which are crucial for improving the structure and functionality of associated devices. Due to its unique photoelectric capabilities, monolayer WSe_2_ has recently received a lot of attention in the fields of atomically thin electronics and optoelectronics. In this work, we focus on the evolution of the temperature-dependent dielectric function (ε = ε_1_ + *i* ε_2_) of monolayer WSe_2_ over energies from 0.74 to 6.40 eV and temperatures from 40 to 350 K. We analyze the second derivatives of ε with respect to energy to accurately locate the critical points (CP). The dependence of the observed CP energies on temperature is consistent with the alternative domination of the declining exciton binding energy as the temperature increases.

## Introduction

Due to its unique photoelectric properties^1–8^, including layer-modulated bandgaps, moderate mobility^2^, a high on–off ratio^9^, and a noticeable spin–orbit coupling effect^1^, the monolayer WSe_2_, a two-dimensional transition-metal dichalcogenide (2D-TMDC) has recently garnered significant interest in the fields of atomically thin electronics and optoelectronics^10–13^. The low dimensional materials have strong Coulomb interaction by reducing dielectric screening and confining electron motion spatially, leading to the formation of highly stable, tightly bound electron–hole pairs known as excitons, characterized by substantial binding energy^14,15^. The utilization of monolayer WSe_2_, whether in its intrinsic form or as part of tailored heterostructures hybridized with other materials, substantially enhances the performance of related optoelectronic devices and imparts a range of unique features to them^9^. The intrinsic optical and dielectric properties of monolayer WSe_2_, typically described by the dielectric function or the complex refractive index^16,17^, exert a strong influence on the performance of these optoelectronic devices. Moreover, these properties often exhibit distinct layer-dependent behavior. Therefore, it is important to thoroughly investigate the dielectric function of WSe_2_ at various temperatures in order to elucidate the underlying physical mechanisms that allow these innovative devices to be created and enhanced.

Photoluminescence (PL)^8^, raman^18,19^, reflection^20,21^, transmission, and absorption spectroscopies are the principal experimental methods used to investigate the optical characteristics of monolayer WSe_2_. These methods provide valuable physicochemical data, such as optical bandgap and absorption properties, however, these measurements depend on sample surface quality and instrumentations therefore we can see spread in reported data^21^. Also, quantity like refractive index (*n*) is obtained mathematically from absorption data via Kramers–Kronig (KK) relation means experimental errors cannot be accessed affecting reliability of the measured data. Spectroscopic ellipsometry (SE) is well known as a powerful optical technique to measure the real (ε_1_) and imaginary (ε_2_) parts of dielectric function (ε = ε_1_ + *i* ε_2_) of materials independently without using KK transformation providing the way to judge measured data^22,23^. As a result, Diware et al.^24^ were able to see Rydberg exciton series even at room temperature using SE. In Ref.^21^, they studied systematically on the temperature dependence of the dielectric function of WSe_2_ monolayers. However, the reflection data and KK relations were used to calculate the dielectric function.

In this report, broad-band temperature-dependent optical properties of WSe_2_ monolayer are measured and analyzed in detail to elucidate the underlaying physical mechanism. A systematic method based on critical point (CP) analysis using the second derivative method is used to extract the characteristics of the optical transitions. We observed eight CPs at room temperature within measured energy range and were able to resolve fourteen CPs at low temperatures due to reduced electron–phonon interaction.

## Experiment details and data analysis

### Sample characterization

The WSe_2_ monolayer sample used in this study was obtained from 2D Semiconductor Inc., which was grown by low pressure chemical vapor deposition over c-cut sapphire substrate^25^. Deposited monolayer shows complete coverage over substrate. Even though quality of the sample was insured by the sample provider, we confirm the quality by Raman spectroscopy and atomic force microscopy (AFM) prior to SE measurements, as shown in Fig. S1 in the supplementary. Due to complete coverage of the monolayer film, we could not find the natural area to measure accurate thickness using normal AFM. Therefore, automated AFM with hard cantilever tip NM-RC from Park Systems Co. was used to make sharp boundary to measure accurate thickness. As shown in Fig. S1a, part of the WSe_2_ monolayer was removed in the form of 2 × 2 μm^2^ with a tip hardness of 350 N/m and set-point force of 0.2 V. The resulting AFM micrograph shows the WSe_2_ thin film surface with grain boundary and a scratch area. The WSe_2_ thin film thickness is 0.77 nm which suggest it is a monolayer. Raman spectra of WSe_2_ monolayer was measured using 541 nm laser excitation with 1 mW power. Two characteristic phonon modes, $E_{2g}^{1}$ (in-plane) and A_1g_ (out-plane) were observed. The separation between these phonon modes is about 9/cm and intensity ration is about 2.0, which are the characteristics of the WSe_2_ monolayer.

#### SE measurements

The experimental configuration used in this work is well described in our previous work^26^. Briefly, the sample was mounted on the cold figure where low temperature is achieved by close-cycle refrigeration. A cold environment was created inside a chamber which was maintained at a pressure of 10^–8^ Torr to avoid unnecessary condensation. Also, the cold chamber was fitted with stress-free fused-quartz window to avoid noise from optical window. A commercial dual-rotating-compensator type (model RC2, J. A. Woollam Co., Inc. at the Multi dimension material convergence research center of Kyung Hee University) ellipsometer is used in this study. The angle of incidence (AOI) is fixed at 68.2°. Dielectric function spectra in the energy range of 0.74 to 6.40 eV were acquired for temperatures from 40 to 350 K. Additionally, SE data at variable AOI of 60°, 65°, and 70° for WSe_2_ and sapphire substrate were pre-measured to avoid fitting correlations, as shown in Fig. S2.

#### Determination of dielectric function of WSe_2_

As measured SE data is called as the pseudo-dielectric function 〈*ɛ*〉 which contains all the information coming from penetration depth of a probing light beam, so generally surface roughness and substrate effects are included in <*ɛ*>. In this work, data were analyzed using a three-phase optical model consisting of the ambient, the WSe_2_ monolayer, and the sapphire substrate. The thickness of WSe_2_ was determined by applying the Cauchy model to the data in the transparent region between 0.74 and 1.5 eV, and the yielded thickness is 0.75 nm, agrees well with our AFM measurement, see Fig. S1a–c, and literature^19,27–29^. As seen from AFM measurements in Fig. S1, WSe_2_ monolayer surface is atomically smooth and uniform, so no need to use a surface-roughness layer in data fitting, which reduces the fitting parameters. Therefore, by fixing the *ɛ* of sapphire and the thickness of the WSe_2_ monolayer, the real and imaginary parts of dielectric function of WSe_2_ are concurrently determined at each wavelength using point-by-point fitting.

#### The critical-point-energy method

To better resolve the overlapping CP structures, second derivative spectra $\frac{{d^{2} \varepsilon }}{{dE^{2} }}$ were obtained using the maximum-entropy method of differentiation with appropriate smoothing^30^. CP parameters were extracted using the standard analytic CP expression^31^

1 $$\frac{{d^{2} \varepsilon }}{{dE^{2} }} = \left\{ {\begin{array}{*{20}c} {A(n - 1)e^{i\phi } (E - E_{g} + i\Gamma )^{n - 2} ,n \ne 0} \\ {Ae^{i\phi } (E - E_{g} + i\Gamma )^{ - 2} ,n = 0} \\ \end{array} } \right.$$

where a CP is represented by the amplitude *A*, threshold energy *E*_*g*_, broadening Γ, and phase *ϕ*, all treated as adjustable parameters. The exponent *n* has the values − 1, − 1/2, 0, and + 1/2 for excitonic, 1, 2, and 3D CPs, respectively. We note that the excitonic lineshape has been used widely for TMDC materials, yielding high quality fit to experimental data^26,32^. For this work, excitonic, 1D, and 2D lineshapes are considered, since 3D is forbidden in 2D materials^33^.

## Result and discussion

**Figure 1:**
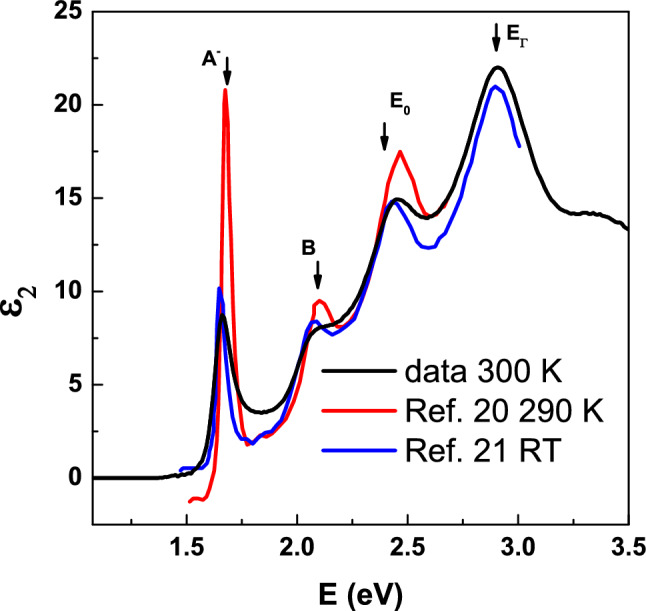
The imaginary part of dielectric function of monolayer WSe_2_ of our data at 300 K (black line) compared with Ref.^20^ (red line) at 290 K and Ref.^21^ (blue line) at RT.

Figure 1 shows a comparative analysis of our findings near room temperature, 300 K, (depicted by the black line) in relation to previously documented data. To improve clarity, we have limited the displayed data to the 1.25–3.5 eV range. Data above 3.5 eV cannot be compared as previous reports have not investigated this region. Data of Refs.^20^ and^21^ obtained via optical reflectance are shown by the red and blue lines, respectively. Notably, the peak positions of the *A* and *B* excitons, located at approximately 1.7, 2.15 eV, are well-defined across all spectra, and their amplitudes exhibit relative consistency. This convergence in amplitude strongly supports the accuracy of our data. Our data demonstrates a gradual decline of the *ε*_2_ value from the peak *A* to zero, extending below the optical band gap. This phenomenon is considered a hallmark of exceptional sample and data quality. The intensity of peak *A* is lower than the previous result. This discrepancy arises because the value immediately preceding peak *A* in the Ref.^20^ is smaller than 0. This may be attributed to a potential error in the K-K transform applied to the reflection data. It is expected to be less accurate than the measurement of *ε*_2_ achieved by ellipsometry, as implemented here and in Ref.^24^. Noting that earlier research involving monolayer WSe_2_ exhibited similar positions of the *A* and *B* exciton peaks. As a result, we confidently assert that the SE data presented in this study accurately represents the dielectric-function values of WSe_2_.

**Figure 2:**
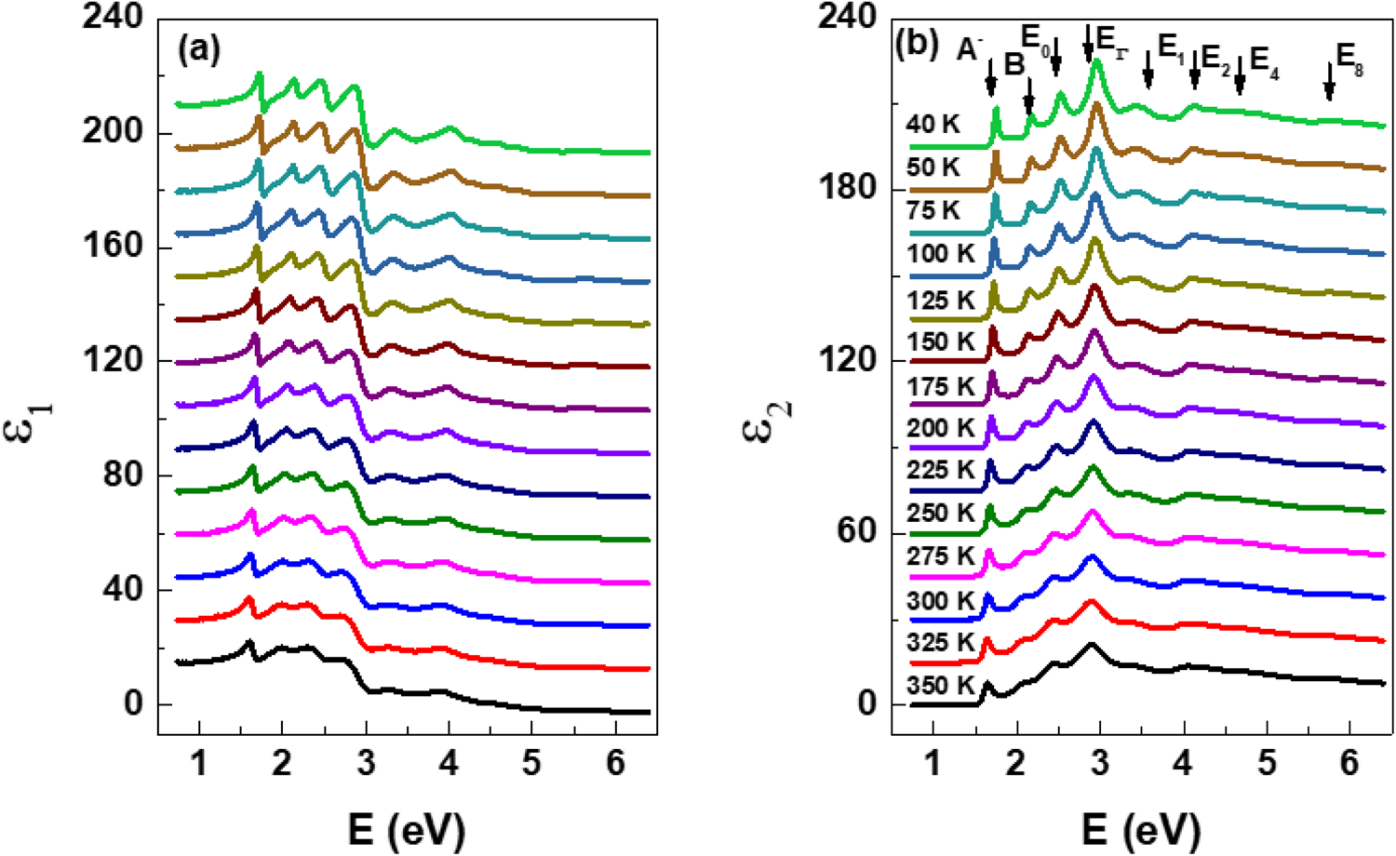
(**a**) Real and (**b**) imaginary parts of the dielectric function of WSe_2_ at temperatures from 40 to 350 K. The spectra are offset by increments of 15 for clarity.

Using the same analytical approach at 300 K, we present the real and imaginary components of the dielectric function for monolayer WSe_2_ across a temperature range of 40–350 K, as depicted in Fig. 2a and b respectively. It is noteworthy that this spectral range lies well below the fundamental bandgap of sapphire^34^ (~ 9.0 eV), so the value of the imaginary part of the dielectric function of the substrate is accurately zero, and the real part exhibits minimal variation. Consequently, we adopt a temperature-independent dielectric function for the substrate, a reasonable approximation under these conditions.

For clarity, the spectra have been offset vertically by increments of 15, and the number of temperature points has been reduced. Within each spectrum we readily discern the presence of 15 CP structures denoted as *A*, *B*, *E*_0_, *C*, *E*_0_ + Δ_0_, and *E*_1-9_. We observe that these CP structures exhibit blue shifts and enhanced characteristics at lower temperatures. This enhancement facilitates the identification of new, smaller CPs, as it mitigates the impact of the strong thermal noise experienced at room temperature. These observed changes can be elucidated by considering the reduction in electron–phonon interactions and the contraction of lattice constants at lower temperatures.

**Figure 3:**
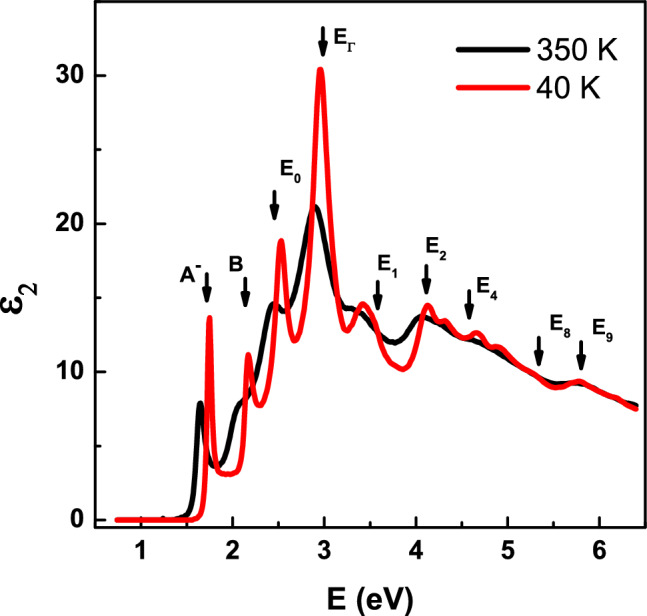
Imaginary parts of dielectric function of monolayer WSe_2_ at 40 and 350 K.

We highlight the changes in Fig. 3, where a comparison of the imaginary part of the dielectric function for the lowest and highest temperatures are shown. We observe differences in the number of peak displacements. At the highest temperature, Peak *E*_1_ is no longer visible, and peaks *E*_4_ and *E*_5_ cannot be distinguished. The peaks *E*_6_, *E*_7_, *E*_8_, and *E*_9_ exhibit similar behavior. It is crucial to note that the qualitative determination of CP energies from the original spectra is challenging due to the pronounced asymmetry of the CP structures, stemming from contributions from transitions across various regions of the Brillouin zone. Therefore, we have employed a standard procedure to determine CP energies at different temperatures, involving the analysis of derivatives.

**Figure 4:**
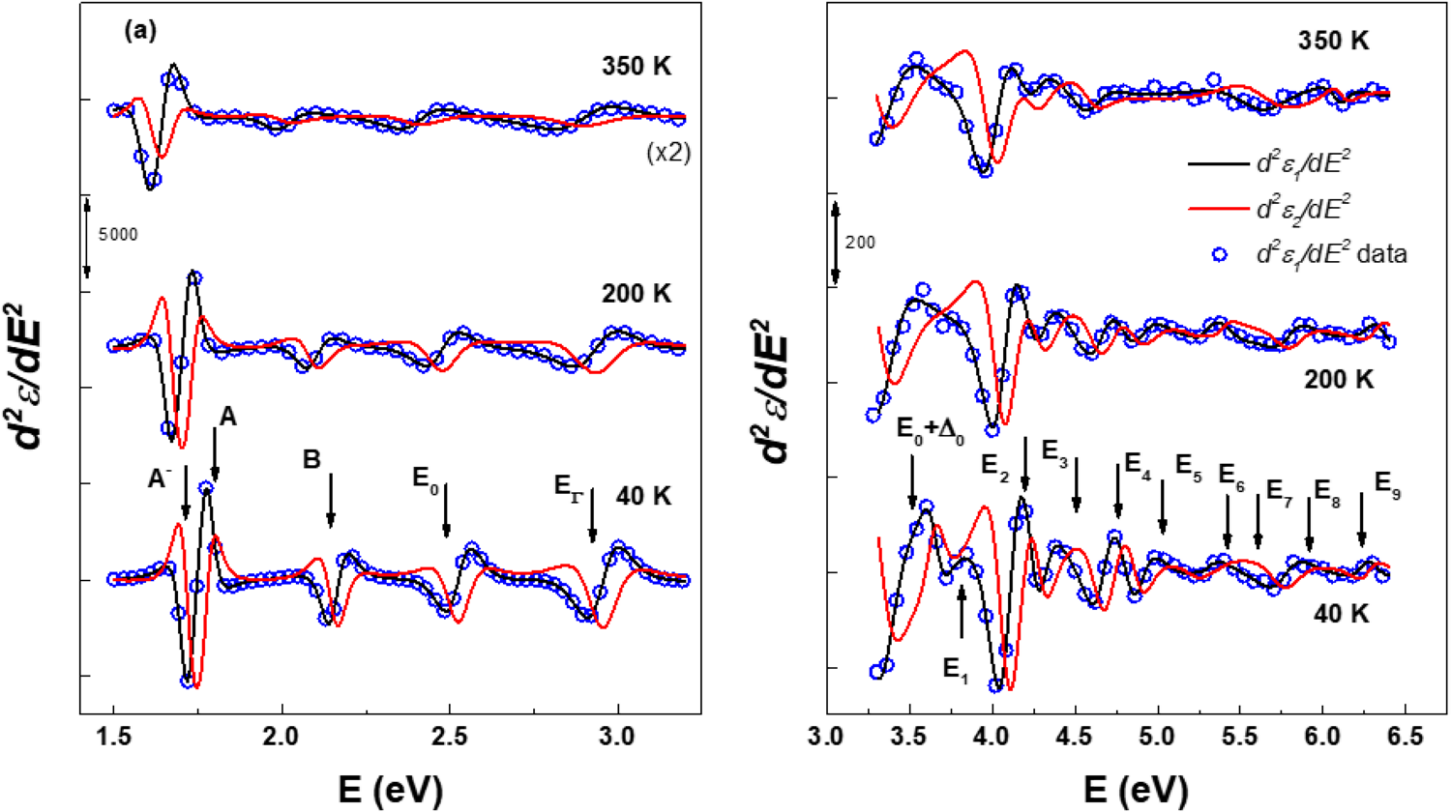
The best fit for $\frac{{d^{2} \varepsilon_{1} }}{{dE^{2} }}$ (black lines) and $\frac{{d^{2} \varepsilon_{2} }}{{dE^{2} }}$ (red lines) from (**a**) 1.5 to 3.6 eV and (**b**) 3–6.40 eV. The data at 350 K in (**a**) is multiplied by two and the data for $\frac{{d^{2} \varepsilon_{2} }}{{dE^{2} }}$ are not shown for clarity.

Figure 4 provides a visual representation of the 2nd derivatives, accompanied by their corresponding best-fit curves. In this figure, open circles denote the 2nd derivatives of measured values for *ε*_1_, while the black and red lines represent the best fits for the real and imaginary parts, respectively. To enhance clarity, the second derivatives of the data for ε_2_ are not shown.

To facilitate comprehension, the spectra have been offset, with increments of 12,500 for Fig. 4a and 5000 for Fig. 4b. As temperature decreases, a clear blue shift in the CP energies becomes evident. At 40 K, the CP structures exhibit significantly sharper characteristics than those at higher temperatures, thereby unveiling the presence of new CPs in region above 3.6 eV. In Fig. 4a, a distinctive pattern emerges within the exciton *A* region. We note that the *A*^-^ trion consistently exhibits a higher dominance compared to the *A* exciton. This recurring phenomenon aligns closely with the material's intrinsic characteristics as an n-type semiconductor. These observations contribute deeper insights to facilitate the accurate design and development of optoelectronic components and devices based on monolayer WSe_2_.

Table 1 presents our results for the CP energies at 40 K and 350 K with a band calculations^35^ and other experimental results obtained by SE as well as results obtained by reflection anisotropy spectroscopy (RAS) and photoluminescence (PL). The results are consistent for the exciton *A* peaks at both low and room temperatures. The previous PL report does not show the trion *A*^-^ peak at room temperature. Our results are in good agreement with RAS^20^ results at low temperatures. In Ref.^25^, authors observed the Rydberg exciton series even at room temperature SE spectra using the same critical-point-energy method presented here. Consequently, CPs may overlap, leading to variations in energy and other CP parameters. Therefore, we would like to emphasize that the combination of low temperature measurements and critical-point-energy method in this work should extract more reliable and accurate CP parameters. As mentioned, our findings align closely with results obtained using other methods. From the Table 1, we can observe that the CP energy differences between the *A*^-^, *A*, and *B* excitons at 40 K and 350 K are similar.

**Table 1:** CP energies (eV) at 40 K and 350 K compared to previously reported data.

CPs energy (eV)	This work		References				
	SE		SE^24^	RAS^20^		PL^8^	Theory^35^
	40 K	350 K	RT	80 K	290 K	RT	
A^−^	1.72	1.60	1.50	1.72			
A	1.77	1.66	1.55	1.75	1.68	1.65	1.5
B	2.15	2.00	1.98				2.0
E_0_	2.52	2.41	2.35				2.4
E_Γ_	2.95	2.90	2.71				
E_0_ + Δ_0_	3.33	3.30					
E_1_	3.65	–					
E_2_	4.09	3.99					
E_3_	4.28	4.15					
E_4_	4.68	–					
E_5_	4.87	4.78					
E_6_	5.27	–					
E_7_	5.35	–					
E_8_	5.80	5.57					
E_9_	6.35	6.06					

**Figure 5:**
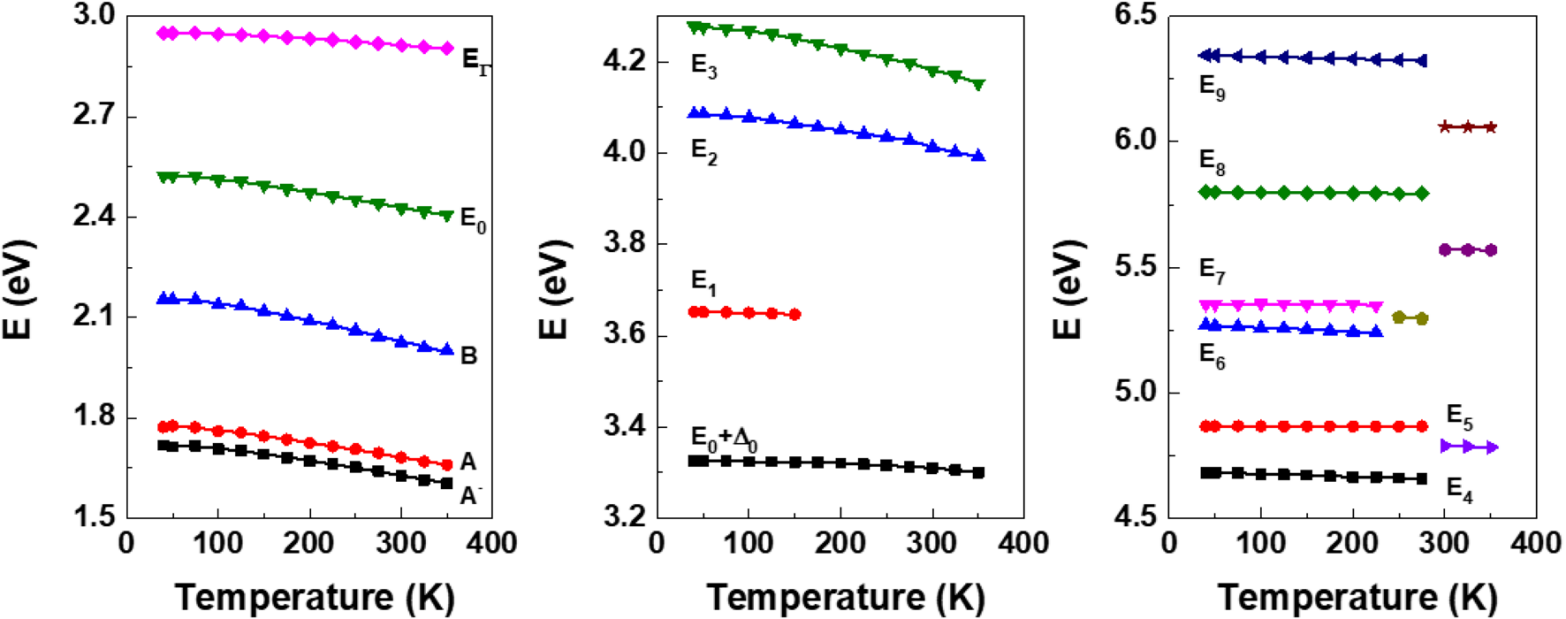
Temperature dependences of the CP energies of WSe_2_.

This similarity in their energy differences is more clearly illustrated in Fig. 5, which shows CP energy values (represented by dots) acquired through the second-derivative analysis. The fit results are depicted as solid lines, obtained using a phenomenological expression that includes the Bose–Einstein (BE) statistical factor for phonons^36,37^:

2 $$E(T) = E_{B} - E_{a} \left[ {1 + \frac{2}{{e^{\Theta /T} - 1}}} \right]$$

Here, Θ represents the mean frequency of phonons, and *E*_*B*_ signifies the interaction strength between electrons and phonons. For other CPs exhibiting negligible curvature in their temperature dependence, we fit a linear equation^35,36^:

3 $$E(T) = E_{L} - \lambda T$$

where *E*_*L*_ is an adjustable parameter and *λ* is the temperature coefficient -*dE*/*dT*. The best fit parameters are listed in Table 2.

**Table 2:** Best-fitting parameters of the temperature dependences of the CPs of monolayer WSe_2_.

CP	E_B_ (eV)		a_B_ (meV)		Θ (K)		E_L_ (eV)		λ (10^–4^/eVK)	
	Value	Error	Value	Error	Value	Error	Value	Error	Value	Error
A^−^	1.79	0.01	72	8	288	21				
A	1.83	0.01	55	6	235	19				
B	2.25	0.01	91	10	268	21				
E_0_	2.59	0.01	60	6	243	19				
E_Γ_	2.98	3 × 10^–3^	33	2	309	14				
E_0_ + Δ_0_	3.43	0.02	101	21	792	63				
E_1_							3.65	0.04	0.48	0.05
E_2_	4.15	0.01	66	10	313	31				
E_3_	4.36	0.01	86	7	313	19				
E_4_							4.69	0.5	1.10	0.03
E_5_							4.87	0.01	0.06	0.00
E_6_							5.28	0.08	1.59	0.06
E_7_							5.36	0.12	0.22	0.08
E_8_							5.80	0.02	0.26	0.01
E_9_							6.35	0.03	0.91	0.02

Additionally, we employed a matching procedure with the Manoogian-Woolley (M-W) expression to analyze the band gap behavior^38^.

4 $$E_{0} (T) = E_{0} (0) + UT^{x} + V\theta \left[ {\coth \left( {\frac{\theta }{2T}} \right) - 1} \right]$$

**Figure 6:**
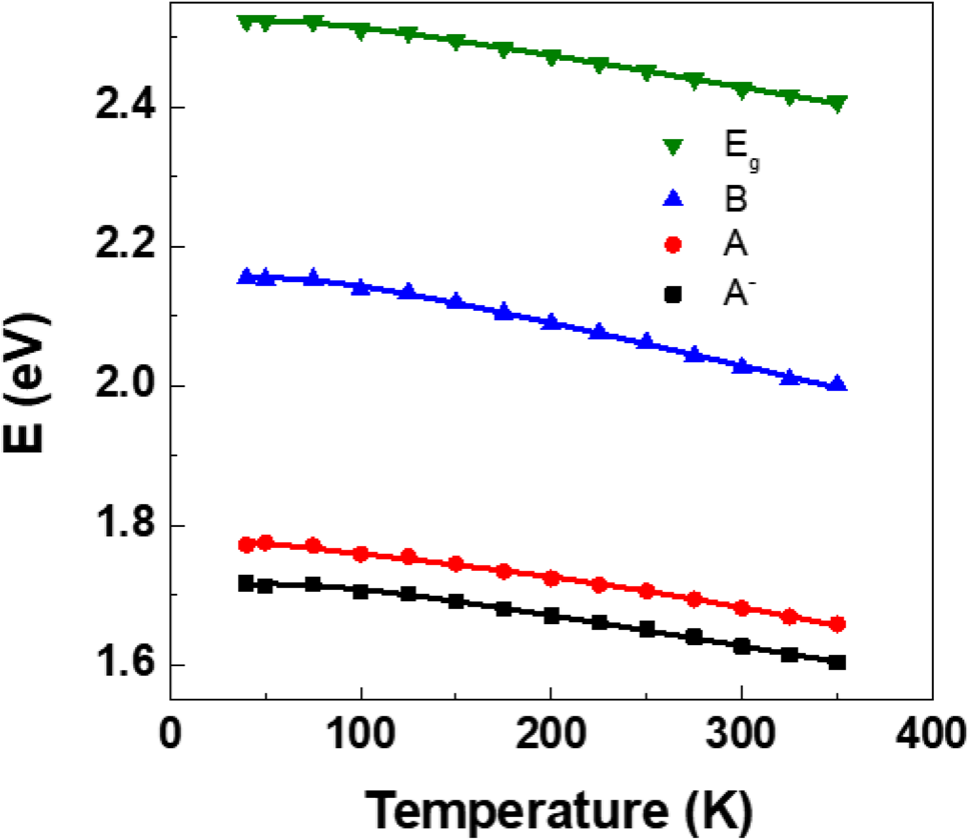
Temperature dependences of the excitons and band gap of WSe_2_ and best fit with MW expansion.

Figure 6 shows our experimental data alongside the best fit obtained by using the M-W. The M-W equation offers insights into the temperature dependence of band gap, where *E*_0_(0) represents the band gap at 0 K, while the second term, *UT*^*x*^ accounts for the lattice dilation. The dynamic component of the energy gap shift in semiconductors is described as *V∙θ∙*coth(*θ/*2*T*), where *V* is an adjustable parameter, *T* is temperature, and *θ* signifies the mean frequency of the total phonon spectrum. The fitting outcomes reveal that *E*_0_(0) = 2.523 eV. The derived value of 1.09 for *x* falls within the range of 0.6–1.2 as documented in the literature^39^. The determined thermal expansion coefficient, *U*, is 3.4 × 10^–4^/K, indicating non-linear expansion, which is in a same order to the linear expansion coefficient of bulk WSe_2_ along the *a*-axis, which stands at 6.8 × 10^–4^/K^40^. However, discrepancy in the absolute values can be attributed to bulk crystal nature and linear expansion, whereas our results are for monolayer and non-linear expansion. Regarding *θ*, which signifies the mean frequency of the total phonon spectrum, the fitting yielded a value of *θ* = 160 K, consistent with the Debye temperature of bulk sample^41^. Table 3 shows the comparison of the thermal expansion constant of bulk and monolayer WSe_2_ obtained from the Raman spectroscopy and X-ray diffraction.

**Table 3:** Thermal expansion of WSe_2_.

	Our data	Ref.^40^^a^	Ref.^41^^a^
	SE	Raman	XRD
α	3.4 × 10^–4^	6.8 × 10^–4^	5.1 × 10^–6^

^a^Bulk WSe_2_.

In Fig. 6, the behavior of the *A*‾, *A*, and *B* transitions exhibit a similar trend with temperature, providing strong evidence that their origins are similar. The energy difference between *A* and *A*‾, referred to as the trion binding energy, and the separation between *B* and *A*, representing the spin–orbit splitting at the maximum valence band at the K valley, appear to be temperature-independent, remaining constant at approximately 54 meV and 382 meV, respectively.

Conversely, the temperature dependence of *E*_1_, which is fit with a linear equation, introduces some uncertainty due to the relatively small amplitude of the *E*_1_ CP, which is evident at only lower temperatures as seen in Fig. 5. This diminished amplitude makes it challenging to accurately detect and fit, leading to the observed uncertainty.

The energy gap between *E*_0_ and *A*, known as the exciton binding energy, is approximately 1.18 eV at 40 K and 1.25 eV at 350 K. This value remains almost constant with temperature, in good agreement with the result reported in previous work^6,20,25^. It is worth noting that Ref. 11 points out that the overlap between the electronic band gap of monolayer WSe_2_ and continuous excitonic CPs makes it difficult to observe the fundamental band gap (*E*_0_) through conventional absorption spectroscopy, even at cryogenic temperatures. However, we not only observe the *E*_0_ band gap as a shoulder-like curvature in the original *ε*_2_ spectrum at 40 K but also systematically investigate its temperature dependence to confirm its authenticity as a consistent CP structure. Additionally, at higher energies, the CPs exhibit a smaller redshift with increasing temperature.

## Conclusions

This study presents a comprehensive analysis of the temperature dependence of the CP energies of monolayer WSe_2_ from 40 to 350 K. We focus particularly on the CP energies, which are derived from the analysis of dielectric function spectra obtained through spectroscopic ellipsometry from 0.74 to 6.40 eV. At 350 K, we determined the energies of eleven CPs. Additionally, four more CPs, *E*_1_, *E*_4_, *E*_6_, and *E*_7_ were discovered at cryogenic temperatures through the analysis of *ε* spectra using critical-point-energy method.

Most CPs exhibit a noticeable blue shift and an augmented structural complexity at lower temperatures. This phenomenon can be attributed to the reduction in lattice constant and the diminishing impact of electron–phonon interactions. The temperature dependence of each CP is effectively modeled either through a linear equation or a phenomenological expression featuring the Bose–Einstein statistical factor. At lower temperatures, we distinctly observe the separation of excitons *A*, *B*, and trions *A*‾. The charged exciton peak *A*‾ dominates the neutral exciton peak *A* at all temperatures. This behavior aligns with the n-type semiconducting characteristics of the material. In addition to the electronic band gaps, we report exciton and trion binding energies, as well as spin–orbit splitting. These findings provide valuable insights that will facilitate the precise engineering of optoelectronic components and devices based on monolayer WSe_2_.

### Supplementary Information

Supplementary Figures.

## Data Availability

The datasets used and/or analysed during the current study available from the corresponding author on reasonable request.
