# Supplementary material for: Temperature dependence of the dielectric function and critical points of monolayer WSe2
Source: Sci Rep. 2024 Jun 12;14:13486. doi: 10.1038/s41598-024-64303-1 (PMC11169377; doi:10.1038/s41598-024-64303-1)
Supplement: Supplementary file 1 — Supplementary Figures. [file 41598_2024_64303_MOESM1_ESM.docx]

Supplementary materials

Temperature Dependence of the Dielectric Function and Critical Points of Monolayer WSe_2_

Xuan Au Nguyen^1^, Van Long Le^2^, Sokhyon Kim^1,3^, Young Duk Kim^1^, Mangesh S. Diware^4^, Tae Jung Kim^1,5,^*, Young Dong Kim^1,^*

*^1^Department of Physics, Kyung Hee University, Seoul 02447, Republic of Korea*

*^2^Institute of Materials Science, Vietnam Academy of Science and Technology, Hanoi 100000, Vietnam*

*^3^Department of Information Display, Kyung Hee University, Seoul 02447, Republic of Korea*

*^4^Advanced Research Center, Parksystems Co. Suwon, Republic of Korea.*

*^5^Center for Converging Humanities, Kyung Hee University, Seoul 02447, Republic of Korea*

**Correspondence and requests for materials should be addressed to T.J.K. (email: tjkim@khu.ac.kr), Y.D.K. (ydkim@khu.ac.kr)*

| a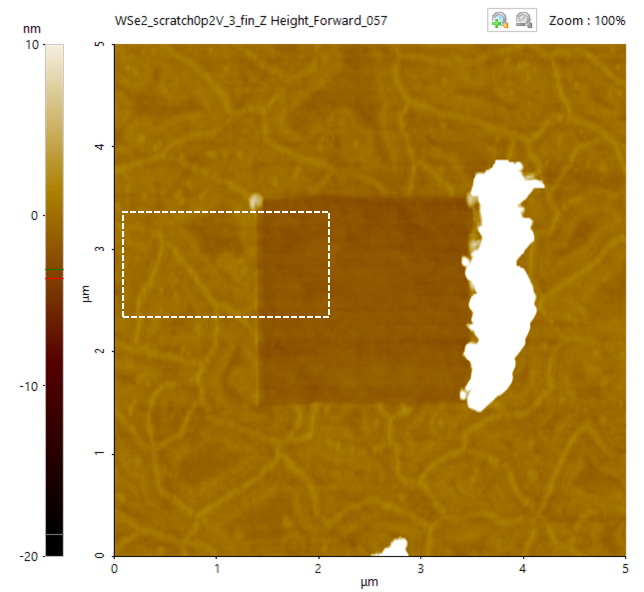 | b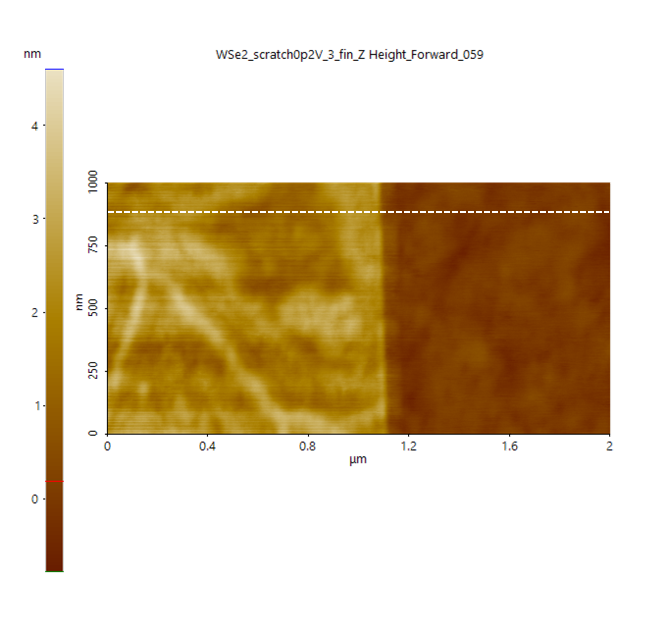 |
| --- | --- |
| c  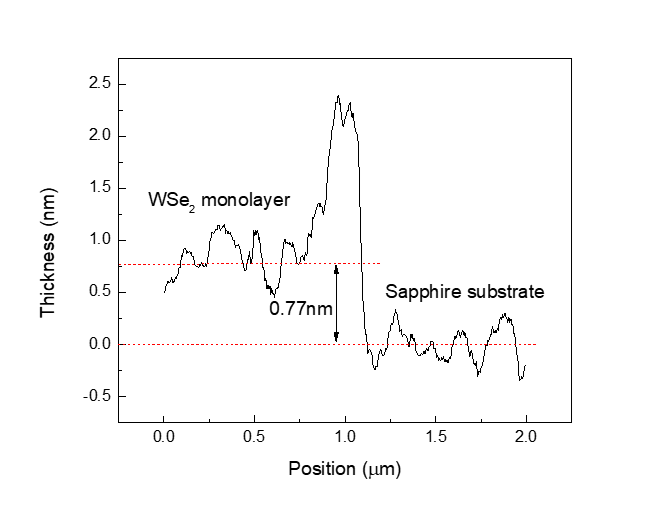 | d   |

Fig. S1. Sample characterization for monolayer WSe_2_

Determining the boundary of the monolayer and the substrate is difficult when the sample covers the full area. In Fig. S1a, we use a cantilevertip NM-RC, K-350 N/m, set point force 0.2 V to create a sample-free 2x2um region. The material WSe_2_ from that region is pushed to one side and has a large thickness (~20-50nm). AFM measurements were performed as shown in Fig. S1.b. Measurement results show that the thickness of the sample is 0.77nm in Fig. S1c. Fig. S1d shows the Raman spectrum of monolayer WSe_2_. The$E_{2g}^{1}$ (in-plane) and A_1g_ (out-of-plane) modes are located at ~251 cm^−1^ and 260 cm^−1^, respectively. The gap between the two modes is about 9 cm^−1^, which is a monolayer characteristic.


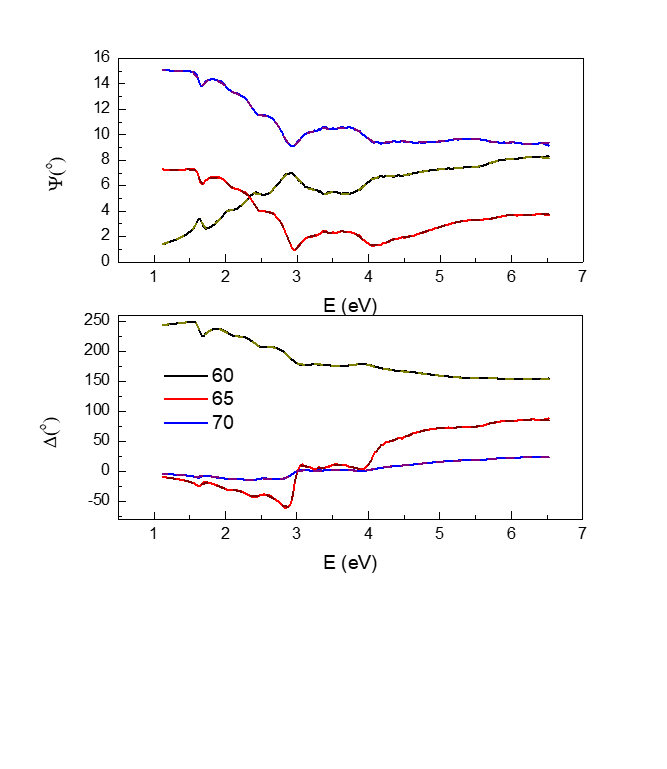


Fig. S2. VASE measurement data of monolayer WSe_2_ at angles of incidence 60º (black), 65º (red ), and 70º (blue) and the best-fit result (solid lines) obtained by point by point fitting. In this fitting we use thickness of 0.75nm determine by Cauchy model in transparent region (under 1.4 eV)
